# Secondary Deposits in Left Supra-Clavicular Glands in Cases of Malignant Disease of Abdominal Organs

**Published:** 1908-07-11

**Authors:** 


### Medicine.

### SECONDARY DEPOSITS IN LEFT SUPRA-CLAVICULAR GLANDS IN CASES OF MALIGNANT DISEASE OF ABDOMINAL ORGANS.

The fact that metastases in the lymphatic glands which lie just behind the clavicular head of the left sterno-mastoid muscle may sometimes be of great diagnostic value in otherwise obscure abdominal cases is better known upon the Continent than it is in this country. There are, of course, very large numbers of cases of abdominal malignant disease in which the glands in question do not become involved at all; but this does not detract from the great value of the sign in the remaining cases. Every year in a large hospital there are one or two patients in whom the diagnosis is cleared up by the presence of enlarged left supra-clavicular glands. The path of infection is probably along the thoracic duct, and there does not seem to be any one form of malignant disease which infects these glands more constantly than another. The primary growth may be in the stomach, the gall-bladder, the pancreas, the suprarenal capsule, the colon, and so on; the discovery of the metastases in the left side of the neck does not afford any indication as to which viscus is the source of the primary trouble; but it does assist in discriminating between malignant and non-malignant abdominal lesions in not a few cases. Moreover, the detection of the glands may save the patient from laparotomy. If they are infected it is clear that the growth has extended beyond all possibility of removal; and if it is desired to find out the nature of the growth it is a comparatively simple matter to remove one of the affected glands, which can then be examined microscopically without any necessity for the performance of a larger operation.

The following instances will show how a knowledge of this point may be of clinical value:—

A man, aged fifty, who regarded himself as being in perfect health, and who had had no single symptom indicative of visceral disease, consulted a doctor about a swelling he had noticed above the sternal end of his left clavicle. He had first observed it a month previously, when it was quite small. It was now about as big as a bantam's egg, and neither painful nor inflamed. It lay deep to the sterno-mastoid muscle, moved freely amongst the subcutaneous tissues, and felt firm and elastic. The patient was not troubled by it in the least, but he had become anxious because it had been enlarging rather quickly. It seemed likely that the gland was the seat of metastatic new-growth, but the most careful examination of the thoracic and abdominal systems did not reveal anything abnormal at that time. It was not until two months later that any decided visceral symptoms developed. These took the form of pneumonia. The patient had what at first seemed to be a typical attack of lobar pneumonia, with signs of consolidation in the lower lobe of the left lung. The abnormal signs did not resolve, however, and in a very short time the patient began to complain of pain in the epigastrium, associated with frequent vomiting and with constipation. Physical examination now revealed a definite abdominal tumour which seemed to be connected with the stomach; at a later date the liver became enlarged, and umbilicated nodules of secondary growth were readily palpable in it. The duration of life was about eight months from the time of appearance of the supra-clavicular swelling.

Another case illustrates the value of microscopical examination of the left supra-clavicular glands. A lady, aged 60, came under observation for pains in the hepatic region. She was a robust woman, who had never ailed until nine weeks previously. Her first symptom then was an acute pain—apparently biliary colic—beginning at the tip of the ninth right costal cartilage, and thence shooting across the epigastrium. At one time there had been transient jaundice. The pains recurred, sometimes with retching. Gall-stones were looked for in the stools, but none were found. The liver was felt two inches below the costal margin,—firm and smooth everywhere except at the region of the gall-bladder, where a hard rounded mass about two inches in circumference was felt. The diagnosis lay between gall-stones and carcinoma of the gall-bladder, and the question of laparotomy was discussed. On further examination, however, it was found that there were three slightly enlarged, firm, freely movable glands above the inner end of the left clavicle behind the sternal end of the left sterno-mastoid muscle. No similar glands were felt on the right side, and there were no other enlarged glands to be felt in the neck or elsewhere.

One of the affected supra-clavicular glands was excised under local anæsthesia. Microscopical sections showed secondary carcinoma. It was, therefore, unnecessary to have an exploratory laparotomy performed, and the patient was spared a severe operation which could not possibly have cured her.

A third case may be of interest, in that it helps to show the path by which the metastases into the left supra-clavicular glands occur—namely, along the course of the thoracic duct. The patient was a middle-aged man, whose urine one July suddenly became dark crimson. This hæmaturia recurred at frequent intervals for several months; there was no particular pain except in the middle of August, when there was a single attack of renal colic. The following January some fresh symptoms developed, of which hæmoptysis was the first, followed shortly afterwards by almost continuous dyspnœa with nocturnal exacerbations. The only abnormal physical signs were some diminution in the vesicular murmur in both lungs, and the presence of fine scattered mucous râles. The sputum became abundant, muco-purulent, and occasionally tinged with blood. No tubercle bacilli nor any other distinctive micro-organisms could be detected in it. Some days before he died the patient spat up a greyish, pulpy mass which looked like a gland, and which proved to be infiltrated with new growth.

At the necropsy it was found that the left suprarenal capsule was the site of a malignant growth which had penetrated into the substance of the kidney, and had thus produced the recurrent hæmaturia. The retro-peritoneal glands had become infected by metastatic deposits, and thence there had been a spread of the growth to the lymphatic glands in the posterior mediastrium. The bronchial glands were also involved, and there was a generalised cancerous lymphangitis of both lungs. Hence the pulmonary symptoms. The mass that was coughed up was a bronchial gland which had reached the interior of a bronchus by a process of ulceration.

The thoracic duct was dissected out in its entirety. From the receptaculum chyli upwards it was firmly adherent to the infected retro-peritoneal and mediastinal glands. Above the level of the roots of the lungs there were two nodules of growth in the wall of the duct itself, and when the tube was traced to its union with the left subclavian vein it was found that there was a nodule of growth at the junction, bulging into the lumen of the vessel immediately below the valve. Close to this there was a typical supra-clavicular gland, enlarged to the size of a small nut, and filled with growth. There was no obvious infiltration of the lymphatic vessels which united this gland witn the thoracic duct, but it seemed clear that the path of infection had been *via* the latter.

